# An Overview of Photocatalytic Membrane Degradation Development

**DOI:** 10.3390/ma16093526

**Published:** 2023-05-04

**Authors:** Mojtaba Binazadeh, Jamal Rasouli, Samad Sabbaghi, Seyyed Mojtaba Mousavi, Seyyed Alireza Hashemi, Chin Wei Lai

**Affiliations:** 1 Department of Chemical Engineering, School of Chemical and Petroleum Engineering, Shiraz University, Shiraz 71557-13876, Iran; binazadeh@shirazu.ac.ir; 2 Department of Nano-Chemical Engineering, Faculty of Advanced Technologies, Shiraz University, Shiraz 71557-13876, Iran; 3 Department of Chemical Engineering, National Taiwan University of Science and Technology, Taipei City 106335, Taiwan; 4 Nanomaterials and Polymer Nanocomposites Laboratory, School of Engineering, University of British Columbia, Kelowna, BC V1V 1V7, Canada; 5 Nanotechnology & Catalysis Research Centre, University Malaya, Kuala Lumpur 50603, Malaysia

**Keywords:** photocatalytic membrane reactor, membrane, nano photocatalyst, drinking water treatment

## Abstract

Environmental pollution has become a worldwide issue. Rapid industrial and agricultural practices have increased organic contaminants in water supplies. Hence, many strategies have been developed to address this concern. In order to supply clean water for various applications, high-performance treatment technology is required to effectively remove organic and inorganic contaminants. Utilizing photocatalytic membrane reactors (PMRs) has shown promise as a viable alternative process in the water and wastewater industry due to its efficiency, low cost, simplicity, and low environmental impact. PMRs are commonly categorized into two main categories: those with the photocatalyst suspended in solution and those with the photocatalyst immobilized in/on a membrane. Herein, the working and fouling mechanisms in PMRs membranes are investigated; the interplay of fouling and photocatalytic activity and the development of fouling prevention strategies are elucidated; and the significance of photocatalysis in membrane fouling mechanisms such as pore plugging and cake layering is thoroughly explored.

## 1. Introduction

Industrial organic pollutants such as hormones, pesticides, pharmaceutically active substances, and personal care products have become a growing environmental concern due to their continuous release into the waterways and subsequent detrimental effects on human health, plants, soil, and aquatic systems [1,2,3,4]. Over a long period, antibiotics in their active form (low doses) and their main components can alter microbial communities in bodies of water [5,6]. Studying water samples of wastewater treatment plants in Beijing, China, revealed high concentrations of tetracycline, sulfonamides, and quinolones [7].

To remove contaminants and lessen their adverse effects on the environment and human health, a number of purification approaches are available, such as chemical coagulation [8], biodegradation [9], flotation [10,11], absorption [12], and adsorption [13,14]. Despite their benefits, conventional procedures have drawbacks, such as their inability to entirely remove contaminants. For instance, there are a number of drawbacks to the adsorption technique used for wastewater treatment, such as the challenging nature of separating the adsorbent from the solution and the high cost of the adsorbent [14,15]. Therefore, it is crucial to develop a novel, incredibly efficient method to eliminate contaminants from wastewater before they enter the environment [16].

The development of innovative green chemical technologies and methods in organic synthesis and environmental converters has emerged as one of the most pressing topics for chemical researchers in recent decades, particularly those working with heterogeneous photocatalysis (HPC). Too much emphasis is placed on exploiting the sun as a renewable energy source, and visible light photocatalysts play a critical role in this regard [17,18]. Progress in photocatalysis, an improved oxidation process, has been rapid in recent years. This technology has gained a reputation for being sustainable and efficient at reducing energy use and harming the environment [19,20]. The membrane separation process is also one of the most common and widely used methods for treating contaminated water. Membrane technology is simple to operate, requires little space, and allows product recovery at high efficiency and high selectivity. Membrane separation is done at normal operating temperatures and does not require phase change [21,22]. Membranes are classified into different types depending on the material, structure, geometry, manufacturing method, and type of process used. Organic, inorganic, and composite materials may be used for production of flat, hollow fiber, or tubular membranes [23,24,25,26]. Based on the substance employed, membrane production is primarily divided into organic (polymeric) and inorganic membranes [27,28]. Inorganic membranes are those that incorporate metals, oxides, or elementary carbon in their structure, such as zeolite, ceramic, and metallic membranes, while organic membranes are those constructed of nonporous polymeric materials, including polyethersulfone, polysulfone, polymethylpentene, cellulose acetate, polycarbonate, polyimide, polyetherimide, polydimethylsiloxane, and polyphenylene oxide [27,29,30]. The separation techniques used in these systems are usually vacuumed filtration or pressure filtration using microfiltration, ultrafiltration, or nanofiltration membranes [31,32]. Polymeric synthetic membranes make up a sizable portion of industrial membranes. Compared to mineral membranes, these membranes are more commonly used because as they are easier to build, less expensive, and more processable [33].

**Figure 1:**
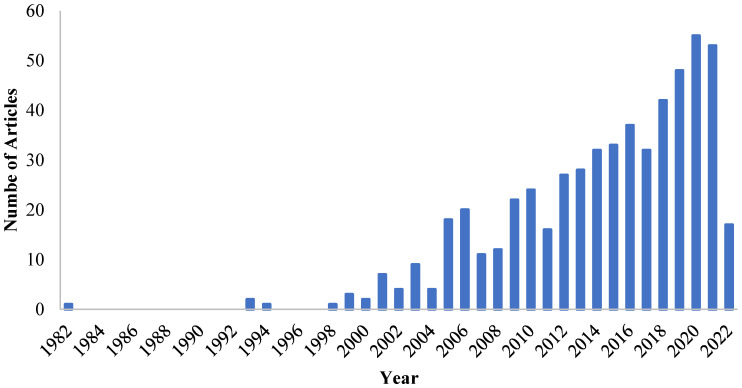
The number of articles published annually on the topic of photocatalytic membrane reactors based on Scopus (searched “Photocatalytic membrane reactor”).

Combining photocatalysis and membrane filtration is a widely used method that can be effective for the removal various contaminants from the aqueous system. Therefore, the design and development of photocatalytic membrane reactors (PMRs) have become a popular and effective method for treating polluted water. The number of articles published annually on PMRs is depicted in Figure 1. PMRs differs from conventional photocatalytic reactors as the photocatalyst remains in the system and treated water selectively permeates through the membrane. PMRs enhance process efficiency, controllability, and stability. They also minimize installation space, energy requirement, and eliminate extra expenditures associated with flocculation, coagulation, and sedimentation [34,35]. The photocatalyst in the PMR system can photodegrade pollutant molecules stuck to the membrane surface using visible light, sunshine energy, and UV radiation [36].

Both polymeric and mineral membranes have shown excellent self-cleaning and anti-fouling properties through photodegradation when exposed to visible light and UV irradiation [37,38]. A photocatalytic membrane may utilize the absorbed energy from the irradiation source and breaks down pollutants that adhere to its surface; thus, such a membrane is self-cleaning and anti-fouling [38].

The main focus of this research is how to use PMRs to clean water and waste water. The photocatalyst, membrane, and light source are the main components of PMRs that are hereby discussed in depth. Membrane fouling is a serious operational concern in slurry-type reactors; thus, its effects on permeate flux and system efficiency are described in length, along with solutions for mitigating the impacts of membrane fouling. Several of the most crucial operational variables that affect PMRs performance are covered. The standards for creating and developing PMRs are provided. Lastly, the most recent developments in the utilization of visible light are also discussed, along with efforts to circumvent some of the inherent drawbacks of PMRs, such as a moderate loss of photoactivity, constrained processing speeds due to mass transfer issues, membrane leakage, and unsatisfactory system lifetimes due to photocatalyst leaching.

## 2. Mechanism of Photocatalysis and Membrane Processes

### 2.1. Photocatalytic Degradation Mechanism

**Figure 2:**
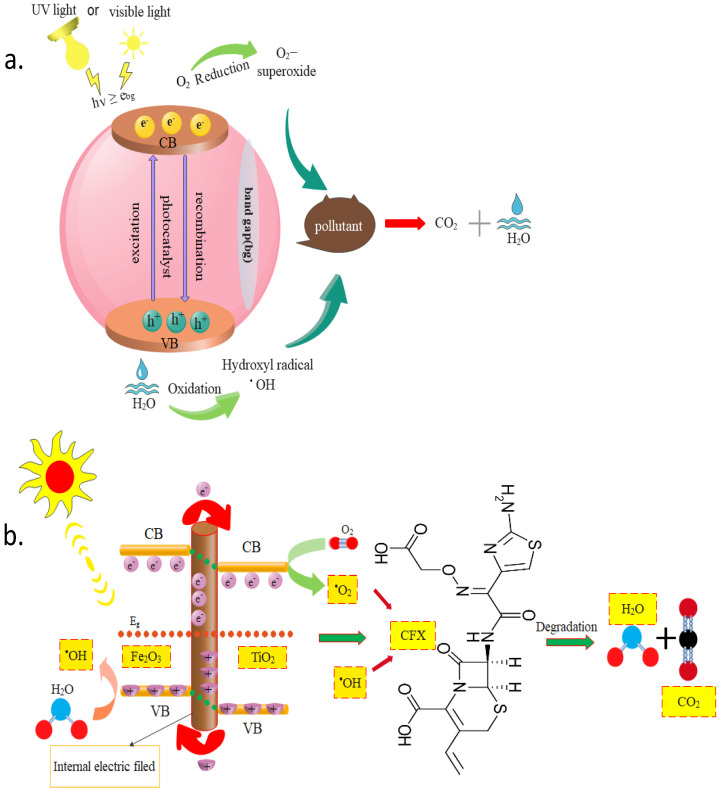
(**a**) Mechanism of photocatalytic degradation of pollutants, and (**b**) mechanism of cefixime photocatalytic degradation via Fe_2_O_3_@TiO_2_ photocatalyst [13]. Adapted with permission from Elsevier. Copyright 2023.

As schematically shown in Figure 2a, degradation reactions are driven by the electrons transferred from the valence to the conduction band. Typically, a photocatalyst’s band-gap energy $(E_{a}$) should be equal to or less than the emitted photon energy [39,40,41]. Electron transfer results in formation of an associated hole ($h_{\mathrm{VB}}^{+}$) in the valence band [42,43]. Electron-hole pairs promote both oxidation and reduction of the adsorbed layer by generation of radicals [17,44]. Radicals are active oxidizing and reducing species that attack to and degrade contaminants in the aqueous solution [45,46]. A substrate reduction potential below that of hole ($h_{\mathrm{VB}}^{+}$) results in the oxidation of substrates in the valence band, whereas a substrate reduction potential higher than that of electron ($e_{\mathrm{CB}}^{-}$) results in the reduction of substrates in the conduction band [47,48]. Hydroxyl radicals (•OH) are the main species responsible for the photodegradation of pollutants. Another oxidant is reactive oxygen species (ROS), such as superoxide oxygen radicals ($O_{2}^{•-}$) [47]. Figure 2b shows photocatalytic degradation of cefixime, as an example of pharmaceutical pollutants, by Fe_2_O_3_@TiO_2_ [13].

### 2.2. Mechanism of the Membrane Filtration Process

Pressure, concentration, or electric potential differences are the driving forces of membrane separation techniques. PMRs operate by pressure and concentration differences. Generally, surface water recycling for non-drinking purposes involves the utilization of ultrafiltration (UF) and microfiltration (MF) membranes. Nanofiltration membranes (NF) with molecular weight cut-offs (MWCO) between 150 and 350 Da are suitable for wastewater treatment and environmental cleanups due to their excellent inorganic ion removal capabilities [21].

**Figure 3:**
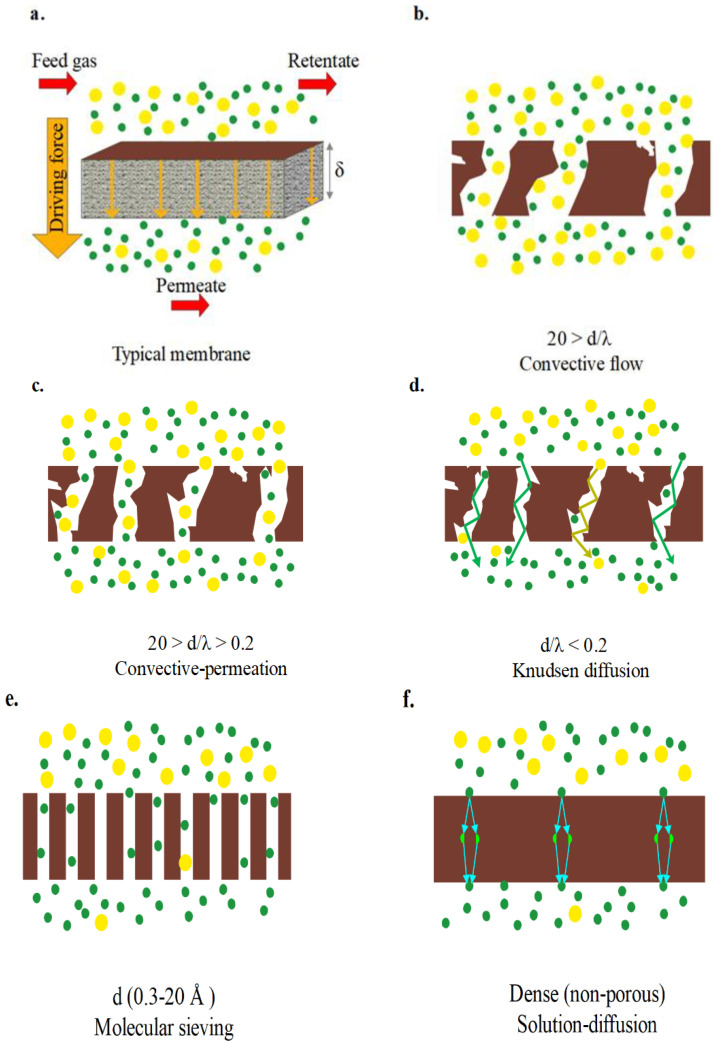
Separation mechanisms in membrane: (**a**) typical membrane, (**b**) convective flow, (**c**) convection–permeation, (**d**) Knudsen diffusion, (**e**) molecular sieving and (**f**) solution–diffusion [65]. Adapted with permission from Elsevier. Copyright 2021.

The contaminated feed solution passes through the membrane under pressure. Membrane in a PMR retains the photocatalyst and pollutants and preferentially permeates water [49,50,51] as depicted in Figure 3a. In photocatalysis conditions at PMRs, polymeris membranes are highly susceptible to (1) abrasion by the photocatalyst and (2) degradation assisted by hydroxyl radicals. Thus, inorganic membranes are preferable due to their superior chemical, mechanical, and thermal resistance. One drawback of inorganic membranes is their cost. A thinner membrane enhances permeate flux due to its lower hydraulic resistance. However, if the membrane is too thin, it may be vulnerable to damage. Average pore size diameter, chemical, mechanical and thermal stability, cost, and lifetime are other important parameters that must be tailored for each membrane application. A list of membranes used for water decontamination is reported in Table 1.

As depicted in Figure 3 there are four main solute transfer mechanisms in membranes. The main transport mechanism for a membrane process is determined by relative magnitude of (1) average pore size diameter (d), (2) size difference of transferable and nontransferable molecules, (3) mean free path of transferable molecule (λ), and (4) pore network structure. According to Figure 3b, presence of large pores in the membrane (d/λ > 20) results in convective flux. Convective flux through membrane accelerates the process; however, convection mechanism is only applicable in PMRs when there is a large size difference between contaminants/photocatalyst and water molecules. If such size difference does not exist, membranes with smaller pore size are required which transport water molecules by permeation. The permeation rate is affected by various factors, including pressure and concentration gradient, size and shape of permeate, and pore size, thickness and chemical structure of membrane. There are two main forces that drive the permeation: hydraulic pressure, which pushes solvent molecules through the membrane, and osmotic pressure, which opposes the flow of solvent due to the presence of dissolved species in the wastewater [60]. The Knudsen diffusion mechanism is dominant when d/λ < 0.2 [61]. Knudsen diffusion occurs only during gas transfer across nano porous membrane; thus, it is useful in removing gases and volatile organic compounds from wastewater [62,63]. Molecular sieves may also be used to purify water [64]. Solution–diffusion is the main transport in dense membranes. Solution–diffusion does not apply to water purification; however, it is the main mechanism for hydrogen transport in Pd-based membranes [65].

During the filtration process, pollutants adhere to the membrane surface and decrease membrane permeability. This phenomenon is calling membrane fouling which is one of the most important challenges of membrane processes [66,67].

Adsorption, accumulation, and precipitation are three mechanisms that may simultaneously occur to produce fouling [68,69]. To improve membrane performance, its surface is modified to maximize its affinity towards the permeating solvent and minimize its affinity towards the fouling agents [70]. Chemical modification, UV irradiation, applied electric field, aeration, and plasma treatment may be utilized to tune hydrophilicity of the membrane surface [71]. Filtration at critical flux has a modest flux drop and minimized irreversible fouling; however, it reduces output. Finally, to maintain the performance of the membrane system, cleaning and maintenance strategies such as backwashing and chemical cleaning are employed routinely during operation [72,73].

#### Characterization of Membranes

**Figure 4:**
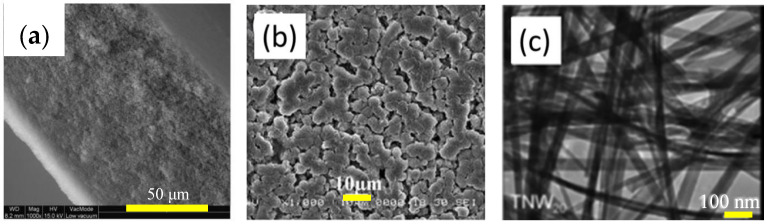
(**a**) Cross-section SEM images of PES membrane [76]. Adapted with permission from Elsevier. Copyright 2008, (**b**) top-view SEM images of PVDF membranes with irregular porous surface [75]. Adapted with permission from Elsevier. Copyright 2007, and (**c**) TEM images showing components of a hierarchical layer of a TiO2 nanowire membrane [77]. Adapted with permission from water and research technology. Copyright 2015.

Membrane production procedure may include phase inversion, interfacial polymerization, and coating. The casting solution and cooling bath utilized in the phase inversion process have a substantial effect on the tortuosity, pore size distribution, pore network. morphology, microstructure, and mechanical properties. Fouling, conditioning, chemical exposure, disintegration, cleaning, the aging process can result in reversible or irreversible alterations to the physical and chemical properties of membranes; thus, membrane characterization is an essential part of membrane manufacturing and maintenance [74]. Characterization techniques such as XRD, SEM, TEM, TGA, DSC, BET, Zeta potential analysis, and FTIR may be employed. SEM images in Figure 4a,b show cross-section PES membrane and irregular porous surface of PVDF membrane, respectively [75,76]. TEM image of Figure 4c shows components of a hierarchical layer of a TiO_2_ nanowire membrane [77].

## 3. Configurations of PMRs

**Figure 5:**
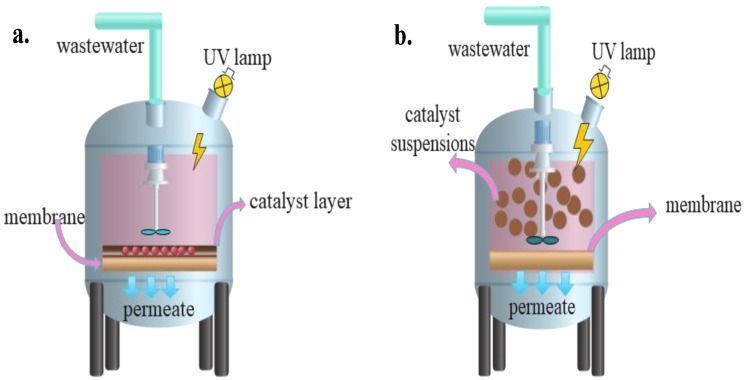
Two different configurations of PMRs (**a**) photocatalyst immobilized in/on the membrane, (**b**) photocatalyst in suspension media.

**Figure 6:**
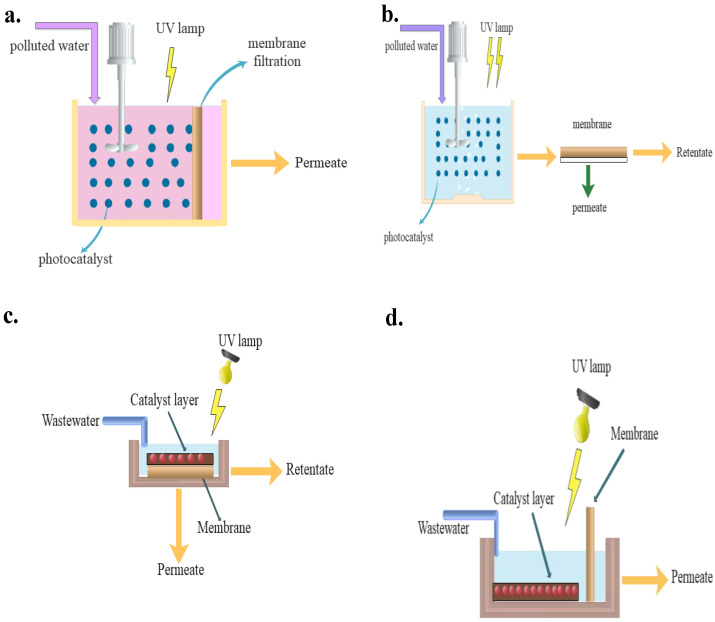
(**a**) Submerged membrane in a slurry reactor, (**b**) Slurry reactor followed by a membrane filtration unit, (**c**) Photocatalytic membrane, and (**d**) Submerged membrane in photocatalytic-coated reactor.

A typical PMR includes a light source, membrane, and photocatalyst [78]. The design and configuration of the PMR dictates process efficiency and controllability. PMRs are classified into two types based on the photocatalyst loading. As schematically illustrated in Figure 5 and Figure 6, suspended photocatalytic membrane reactors (SPMR) are those in which the photocatalyst is suspended, whereas immobilized photocatalytic membrane reactors (IPMR) are those in which the photocatalyst is fixed on a carrier material such as quartz, stainless steel, glass, limestone, or zeolite [79]. When a photocatalyst is immobilized on a support, the active surface available to solution particles is drastically decreased, resulting in a loss of photoactivity [80]. The active surface increases significantly when the photocatalyst is suspended; however, after detoxification, the photocatalyst particles must be separated from the treated water. Table 2 shows the numerous applications of PMRs in wastewater treatment processes.

Benefits of PMRs over traditional photoreactors include: (1) the ability to regulate the residence inside the reactor; (2) continuous operation; (3) the containment of the contaminants and photocatalyst within the reaction environment; (4) enhancing process efficiency and stability; and (5) reduced reactor volume and operating costs [36,42,81].

**Table 2:** Application of PMRS in wastewater treatment process.

Photocatalyst	Pollutant	Type of PMR	Photocatalyst Dosage (wt%)	Pollutant Concentration (mg·L^−1^)	Light Source	Time (min)	Degradation (%)	Ref.
P-doped g-C_3_N_4_ (PCN) and coated on an Al_2_O_3_ substrate	phenol and methyl blue		10	-	visible	-	92 and 90	[82]
MIL-88B(Fe) and coated onto an Al_2_O_3_ substrate	phenol		-	-	visible	-	-	[83]
immobilized N-doped TiO_2_	diclofenac	SPMR	-	-	visible	-	-	[84]
MIL-53(Fe)/PVDF mixed-matrix membrane	tetracycline	IPMR	5	-	UV	-	93	[85]
UVA/TiO_2_-MF	oxytetracycline	Suspended vs immobilized TiO_2_-P25	-	5	visible	30	>90	[86]
polysulfone/H_2_O_2_-g-C_3_N_4_ mixed matrix membrane	humic acid	-	10	-	visible	-	93.5	[87]
NH_2_-MIL125(Ti) MOF	methyl blue	immobilized and suspended	2	-	UV	-	60 and 97	[88]
TiO_2_	ketoprofen	SPMR	-	10		-	61	[79]
TiO_2_-WO_3_/PANI	Cr (VI)	SPMR	5	-	Visible	60	98.5	[89]
TiO_2_/UV-A	nitrate	SPMR	-	-	UV	-	65–90	[90]
TiO_2_	ketoprofen	SPMR	-	10	-	-	75	[79]
Sb_2_O_3_/CuBi_2_O_4_	methyl blue	SPMR	10	10	Visible		94.6	[91]
ZnO/WO_3_	phenol	SPMR	-	30		7	92.5	[92]

## 4. Photocatalytic Degradation of Pollutants

Many organic and inorganic substances, especially toxic or refractory substances are resistant to biological degradation. After the discovery of the photocatalytic splitting of water in 1972 by Fujishima and Honda [93], scientists and researchers turned their attention to semiconductor photocatalysts that could destroy resistant contaminants that were difficult or impossible to remove by other methods [94]. Photocatalytic degradation has emerged as one of the most sustainable, energy efficient, cost-effective and non-hazardous and environmentally friendly processes for contaminants removal from water which uses light as the energy source [95]. The catalyst’s photonic activation mode, which replaces thermal activation, is the primary distinction between photocatalysis and traditional catalysis [96]. Photocatalysts do not contain heavy metal and do not require strong oxidants/reducing agents for activation. Photocatalysis degradation products are harmless [47].

### 4.1. Photocatalytic Degradation of Pharmaceutical Compounds

**Figure 7:**
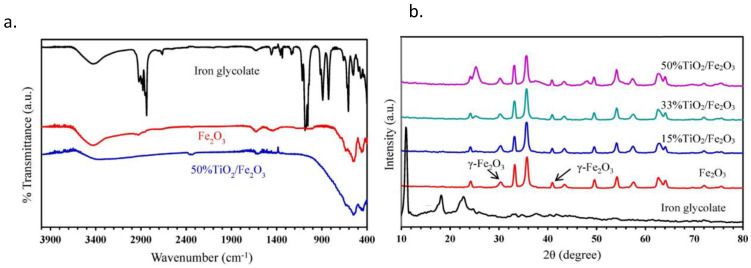
(**a**) FTIR spectra of iron glycolate, pure Fe_2_O_3_ microflowers and 50%TiO_2_/Fe_2_O_3_ core-shell and (**b**) XRD patterns for iron glycolate, pure Fe_2_O_3_ microflowers and TiO_2_/Fe_2_O_3_ core-shells [99]. Adapted with permission from Elsevier. Copyright 2017.

pH is one of the most vital factors that affects photodegradation efficiency. The efficiency of TiO_2_ and ZnO nanoparticles in removing acetaminophen from the water was studies by Ahed et al. [97]. The outcomes of the study demonstrated that ZnO was more effective at eliminating acetaminophen. Ahed et al. proved that pH is an important factor in photodegradation. Their synthesized ZnO photodegraded 97% of acetaminophen at pH = 9 in 1 h of exposure. They demonstrated that at neutral pHs, photocatalytic degradation rates are higher than at acidic pHs. Sabouni et al. [98] investigated the elimination of progesterone, ibuprofen, and naproxen using ZnO photocatalyst. They studied the initial concentration of pollutants and the photocatalyst loading. They found that ZnO photocatalyst is quite efficient in eliminating all three pollutants. They reported progesterone, ibuprofen, and naproxen as having a degradation efficiency of 92.3%, 94.5%, and 98.7%, respectively. The degradation of paracetamol using TiO_2_ and Fe_2_O_3_ photocatalysts was studied by Abdelwahab et al. SEM, TEM, XRD, FTIR, Raman spectroscopy, and VSM analyses were used to characterize the synoecized photocatalysts. Their results revealed that paracetamol degradation increased when TiO_2_ loading in the TiO_2_/Fe_2_O_3_ composite was increased [99]. The FTIR spectra of iron glycolate, Fe_2_O_3_, and 50% TiO_2_/Fe_2_O_3_ reported by Abdelwahab et al. are displayed in Figure 7a. The peaks at 3432 cm^−1^ and 1618 cm^−1^ are attributed to the O-H stretching and bending vibrations of adsorbed water or EG, respectively. Peaks between 2850–2950 cm^−1^ are indicative of C-H vibrations. Between 1120 and 1470 cm^−1^, the CH_2_ bending vibration peaks could be seen. C-O stretching vibrations were responsible for two sharp peaks at about 1050 and 1085 cm^−1^. Peaks of the Fe-O stretching vibration were seen at the 467–660 cm^−1^. All of the peaks related to the glycolate moiety have disappeared in the Fe_2_O_3_ FTIR spectrum, which was obtained after the iron glycolate was calcined at 350 °C [99].

To examine the crystalline structure and phase purity of the prepared sample, the XRD patterns photocatalysts synthesized by Abdelwahab et al. are reported at Figure 7b. When the iron glycolate sheets are stacked, a prominent low-angle diffraction peak at 11° is observed. The diffraction pattern of the Fe_2_O_3_ product created by calcining the iron glycolate at 350 °C shows that the main diffraction peaks match with the α-Fe_2_O_3_ rhombohedral structural pattern. However, two peaks at 2θ of 30 and 43° that are either indicative of Fe_3_O_4_ or γ-Fe_2_O_3_ show that the acquired sample could be made up of α-Fe_2_O_3_ and other iron oxide crystal forms. the relative magnitude hematite (α-Fe_2_O_3_) and maghemite (γ-Fe_2_O_3_) and/or magnetite (Fe_3_O_4_) are 70% and 30%, respectively. Furthermore, the new peaks at 2θ of 25.2, 37.4, 48, and 54° of the TiO_2_/Fe_2_O_3_ samples can be correlated to the (101), (004), (200), and (105) planes of anatase TiO_2_, demonstrating that the TiO_2_ crystallites were placed onto the magnetic core Fe_2_O_3_. The average crystallite size of TiO_2_, calculated by Scherrer’s equation is 12 nm for the anatase (101) peak [99].

Table 3 shows the photocatalytic degradation of different pharmaceuticals. Clearly, heterogeneous photocatalysts with a variety of nanostructures can effectively degrade pharmaceuticals in aqueous solutions.

### 4.2. Photocatalytic Degradation of Dye Compounds

Dyes are routinely used in the textile, food, beverage, printing, and pharmaceutical industries. These compounds may be harmful and cancerous will barricade sunlight from reaching water bodies, impacting natural aquatic processes such as photosynthesis and other biodegradation operations [112,113]. Long-lasting and non-degradable colored pollutants must be removed before entering the environment since their entry leads to the aquatic ecosystem becoming toxic and dangerous to humans [114]. The photocatalytic process has been suggested as one of the more successful methods. In photocatalysis, the degradation is begun with OH radicals breaking the azo bond (-N=N-), which is one of the weakest chemical bonds in the dye molecules’ chemical structure [115]. The process’s intermediates will then undergo a radical chain reaction with the oxygen molecules, finally breaking down to produce water and carbon dioxide [115]. In recent years, numerous studies on the removal of contaminants from actual wastewater have been conducted. Table 4 illustrates the photocatalytic degradation of a variety of dyes.

### 4.3. Photocatalytic Degradation of Hydrocarbons

Industrial use of hydrocarbons inevitably contaminates natural waters through improper disposal or leaching from landfills, spills, or leaks in underground pipes. Hydrocarbon contaminants endanger human health if they enter drinking water. The presence of these contaminants in water hinders light penetration into the water and affects the diffusion/solubility of gases required for aquatic plant respiration which ultimately leads to plant death; therefore, it may affect the food supply chain.

Schnabel et al. [127] used semiconductor titanium dioxide to remove hydrocarbons. This study demonstrated that a variety of photocatalyst designs, when exposed to ultraviolet (UV) light, can remove the non-polar material in diesel fuel. They reported that the floating foam glass catalyst with TiO_2_ coating reduces the concentration from an initial concentration of 668 mg/L to 329 mg/L in 16 h. The contaminant concentration is reduced by 401 mg/L and 55 mg/L when glass fiber and steel grit was used, respectively [127].

Nirmala Rani et al. [128] studied the elimination of three polycyclic aromatic hydrocarbons (PAHs) in mixed or separate states using titanium oxide photocatalysts and MS membranes under UV irradiation in a PMR. They reported the degradation efficiency of 100, 94.1, and 97% in an aqueous mixture containing 1000 µg /L phenanthrene (PHE), 5000 µg/L naphthalene (NAP), and 1000 g/L acenaphthene (ANA), respectively, after 180 min UV irradiation at photocatalyst loading of 0.5 g/L. When the compounds were used as a sole compound, the elimination percentages of PHE, NAP, and ANA were 99.3, 92.8, and 95.3, respectively, under similar operating conditions [129]. The photocatalytic degradation of several hydrocarbons is reported in Table 5.

### 4.4. Photocatalytic Degradation of other Pollutants

Photocatalyst have been used to degrade pollutants listed in Table 6. Pitchaimani Veera Kumar et al. [139] employed zinc oxide nano stars (ZnONSt) coupled with Ag and Pd to photocatalytically degrade herbicides and pesticides. Ag at ZnONSt and Pd at ZnONSt photocatalysts accelerated the degradation of existing pollutants as they facilitate the interfacial charge transfer process [139]. In order to remove the herbicide ametrine, Rodrigo Pereira Cavalcante et al. [140] utilized a titanium dioxide photocatalyst. They were able to completely remove the ametrine after 60 min irradiation of simulated sunlight (using a 1000 W Xenon lamp), and then they used 0.4 g/L of photocatalyst to detoxify the solution. Samsudin et al. [141] utilized BiVO_4_/g-C_3_N_4_ integrated with Pt to purify poultry during sun light exposure. The as-synthesized photocatalyst demonstrated 93.5% COD removal from the starting concentration of 2152 mg/L in 3 h. The photocatalyst showed strong recyclability and photostabiliy.

## 5. PMRs

### 5.1. Operating Factors and Limits of PMRs

#### 5.1.1. Operating Mode

Both dead-end and cross-flow PMRs can be used in photocatalytic systems. In the dead-end configuration, the whole stream is filtered by passing through a membrane (permeate). As a consequence, the concentration of the nontransferable components rises, resulting in creation of a filter cake on a membrane’s surface, as well as a reduction in membrane permeability and photocatalytic efficiency. In the absence of turbulency, e.g., stirring, there will be insufficient contact between the contaminants, photocatalyst, and the light source [152]. Wang et al. [153] investigated a novel photocatalytic membrane created via pressure-driven filtration load with a ZnO/N-g-C_3_N_4_ composite via glutaraldehyde as a crosslinker. SEM, XPS, and FTIR were employed to verify the photocatalyst loading on membrane surface. They found that in both immersion and filtration models, the photocatalytic capabilities of the ZnO/N-g-C_3_N_4_ composite membrane were effective for the decomposition of tetracycline, ofloxacin, and ciprofloxacin under visible light (>420 nm). For tetracycline at 5 mg/L and 10 mg/L concentrations a ZnO/N-g-C_3_N_4_ loading of 1.12 g/cm^2^ resulted in 100% and 80% degradation, respectively. They concluded that a prolonged reaction time on the membrane surface, low trans-membrane pressure (0.005 MPa), and narrow membrane size were advantageous for the elimination of antibiotics in the filtration processes.

#### 5.1.2. Photocatalyst Type and Characteristics

Key parameters that significantly affect photocatalytic efficiency include the type of photocatalyst, the photocatalyst’s physicochemical properties (band gap energy, particle size distribution, crystallographic structure, and chemical makeup), and the photocatalyst’s concentration in the reacting environment. As mentioned previously, photons by energy equal to or greater than the band gap energy can be absorbed in photocatalytic activities, resulting in the creation of electron-hole pairs. However, photocatalysts that require visible light to function are more intriguing, and it has become a challenge for PMR systems to use a light source that is both environmentally friendly and economically viable [154]. For example, TiO_2_-supported photocatalyst is the most frequently employed in PMR in suspended form due to its great photochemical stability in aquatic solutions, robust catalytic activity, reasonably long lifespan of electron-hole pairs, low cost, and low toxicity. This material is inactive when exposed to visible light. As a result, TiO_2_ can only absorb around 5% of the solar radiation that is in the UV spectrum [155]. Ahmad et al. [156]. stated that a developed composite ceramic membrane may benefit from a synergy of dead-end filtration and cross-flow filtration while being subjected to intermittent UV irradiation in order to efficiently prevent membrane fouling. To remove organic dye impurities in a photocatalytic membrane reactor, a partly coated TiO_2_ (pc-TiO_2_) layer was made with the assistance of cheap polyvinyl chloride (PVC) to make gaps in a porous Al_2_O_3_ membrane substrate. Their study revealed that the pc-TiO_2_/Al_2_O_3_ composite membrane has superior water flux and anti-fouling capabilities compared to the uniformly coated TiO_2_/Al_2_O_3_ (UC-TiO_2_/Al_2_O_3_) membrane. The photocatalytic activity of UC-and pc-TiO_2_/Al_2_O_3_ composite membranes was significantly enhanced during cross-flow membrane filtration in comparison to that of the Al_2_O_3_ bare membrane substrate.

#### 5.1.3. Light Source

When light is shone on photocatalysts, photons with energy higher than or equal to the band gap are absorbed, valence band electrons are shifted to the conduction band. The oxidation and reduction reactions that occur are due to the production of electron-hole pairs [4]. Consequently, the kind and intensity of light have a significant impact on the performance of photocatalysis [157].

The sol-gel procedure and the dip-coating may be used to create a nanostructured TiO_2_ film from titanium tetraisopropoxide. Sol-gel nanostructured TiO_2_ (anatase phase) film was investigated for its photocatalytic degradation of azithromycin to determine the most efficient degradation pathway for application in wastewater treatment. At the pH of 10 and UV-C irradiation maximum degradation was achieved. The LED irradiation source with emission wavelength of 365 nm was not as efficient as the UV-C lamp. The LED bulb, however, may be a “real-world” option due to its low price, high energy efficiency, and low environmental impact [158]. Shang et al. [159]. investigated the antibacterial activities of TiO_2_ photocatalysts under various light sources, exclusively under visible light. They discovered that by doping metal ions and nonmetal ions on TiO_2_ and compounding with polymers, they could increase the photocatalytic activity to the visible light region, improve the surface characteristics, and enhance the contact area with bacteria. Reactive oxygen species (ROS) and hydroxyl free radicals damage the cell membrane, DNA, and enzymes.

### 5.2. Degradation of Pharmaceutical Compounds via PMR

Pharmaceutical compounds are structurally complex and environmentally stable. They typically contain abundant aromatic rings. Thus, conventional wastewater treatment methods cannot effectively remove them. Hence, the use of PMRs has become a popular solution for removal of pharmaceutical compounds from water. Fang et al. [153] synthesized a ZnO/N-g-C_3_N_4_ composite and immobilized it on a commercial polymer membrane via GA as the crosslinker to breakdown antibiotics under visible light in a PMR setup. Additionally, the ZnO/N-g-C_3_N_4_ composite photocatalytic membrane properties were evaluated using the immersion model and the filtration model. The amount of ZnO/N-g-C_3_N_4_ loading and GA concentration were significant for the photocatalytic abilities of composite membranes, according to an immersion model. The outcome demonstrated that the filtering model results in a greater antibiotic decomposition at longer photocatalytic reaction time, that could be attained by narrow membrane pore sizes, low TMP, and reduced flow. Using a similar photocatalytic membrane fabrication procedure using pristine membrane with MWCO of 50 kDa, improved TC degradation (71.7%) was obtained by the immersion approach which is attributed to the decreased flux and increased retention time. The Photocatalytic membrane degradation of pharmaceutical compounds is shown in Table 7.

### 5.3. Degradation of Dye Compounds via PMR

Synthetic paint is one of the most abundant pollutants in sewage and effluent of industrial plants. The most commonly investigated dyes are rhodamine B, methylene blue, and methyl orange. Conventional wastewater treatment methods are inefficient in removing dyes. Photocatalytic technology is one of the most successful approaches suggested for dye removal.

Dzinun et al. [180] create a TiO_2_-PVDF photocatalytic membrane by addition of different loading of TiO_2_ nanoparticles on a PVDF membrane for methylene blue removal. They characterized the resulting photocatalytic membranes with FE-SEM, EDS, and AFM. The results of their custom-designed PMR demonstrated that adding TiO_2_ nanoparticles to the PVDF membrane speeds up methylene blue removal from waste water. Kolesnyk et al. [181] studied the impact of g-C_3_N_4_ loading on a commercial PVDF membrane on rhodamine-B removal. The highest removal rate occurred in the alkaline medium. In this study, the membrane lost approximately 15% of its pores after five cycles (a total of 50 h). Yu et al. [163] synthesized a PSF membrane coated with g-C_3_N_4_ and TiO_2_ nanocomposites to investigate the removal of sulfamethoxazole. They reported that sulfamethoxazole was converted into seven other non-toxic substances using their custom-designed PMR with sun light irradiation [163]. Horowitz et al. [179] investigated the efficiency of Al_2_O_3_ coated membranes with pore sizes of 200 and 800 nm for carbamazepine removal at different operating conditions. It was observed that the PMR efficiency under UV irradiation is much higher than that under visible waves. They also reported that the contaminant removal rate increases with temperature. Ma et al. [182] investigated humic acid removal from wastewater using TiO_2_/Al_2_O_3_ photocatalyst and membrane microfiltration processes. They found that light intensity significantly affects humic acid removal. A summary of dye degradation via PMRs is reported in Table 8.

### 5.4. Degradation of Hydrocarbons via PMR

Rani et al. used a membrane photocatalytic reactor containing suspended TiO_2_ photocatalytic particles for naphthalene removal. They studied the impact of different operating parameters such as the initial concentration of naphthalene (5–25 mg/L), photocatalyst loading (0.1–0.9 g/L), and pH (3–9) on naphthalene removal rate. The maximum naphthalene removal by separate photocatalysis and membrane was 76.8% and 49.1%, respectively, while a naphthalene removal of 90.2%. could be achieved by PMR [183]. Batch PMRs functioning in the dead-end mode was designed by Moslehyani et al. [184] which could to eliminate 99% of the hydrocarbons from sludge after 2 h. Ag-TiO_2_-coated alumina membrane in a dead-end configuration to degrade rhodamine rate of 1.007 mg/m^2^h^1^ [185]. Despite the promising outcomes, the researchers emphasized that the dead-end process leads to the buildup of separated substrates on the membrane surface and ultimately forms a cake layer, which decreases photocatalytic efficiency. A summary of hydrocarbon degradation via PMRs is reported in Table 9.

### 5.5. Degradation of Other Pollutants via PMR

Pollutants such as toxins, detergents, and heavy metals that do not fall into the above categories are also treated by PMRs which can be seen in Table 10. For example, it can be seen from Table 10, 99.9% of toxic hexavalent chromium (Cr (VI)) at an initial concentration of 10 mg/L could be removed by PMR containing Chitosan-sodium alginate/Fe-doped WO3 photocatalyst and PES membrane after 240 min using a 300 W Xe light source [186].

**Table 8:** Review on Photocatalytic Membrane degradation of dye compounds.

Pollutant	Pollutant Concentration (mg/L)	Photocatalyst/Synthesis Method	Membrane/Pore Size (µm)	Light Source	Time (min)	Degradation (%)	Ref.
Phenol	50	TiO_2_-GO/modified Hummer’s	PVDF, PAA/0.45	100W UV-C lamp		60	[187]
RhodamineB	10	CNTs/MCU-C_3_N_4_/GO	PVDF	300 W Xe lamp,		98.31	[188]
RhodamineB	-	TiO_2_	PVDF/0.08–0.2	3 UV-C lamps		95	[189]
Eosin yellow	100	N,Pd co-doped TiO_2_/	Polysulfone	500 W Xenon lamp	180	92	[190]
Phenol	5	g-C_3_N_4_/CNTs	Al_2_O_3_/0.297	300 W Xe lamp	60	94	[191]
Methylene blue	1	TiO_2_	Al_2_O_3_/0.02–0.2	UV-LED		80	[192]
Methylene blue	-	ZnWO_4_/NiAl-LDH/Hydrothermal	PVDF/0.45 for pure PVDF		120	93.97	[193]
Methylene blue	500	Co/PC/g-C_3_N_4_		Xe Lamp 300 W	360	99	[194]
Methylene blue	1	NbCxOy/NbOx/g-C_3_N_4_	-	-	480	100	[195]
RhodamineB	10	TMPyP/SPSf/non-solvent-induced phase separation	PES	300 W Xe Lamp	180	93.4	[196]
Methylene blue	10	RGO/PDA/TiO_2_/ Hummer method	CA/0.3–0.52		150	80	[197]
Phenol	10	O-g-C_3_N_4_	PES/0.125–0.188	30W UV lamp,	120	35.78	[198]
RB5	30	F$e^{3+}$ doped ZnO	-	artificial sunlight (D65, 72W)	180	98.34	[199]
Methylene blue, RhodamineB, and methyl Orange	20	TiO_2_/ hydrothermal	PPS/0.185	300 W Xenon lamp	90	RhodamineB 99.56, methylene blue 98.05, methyl Orange 93.18	[200]
Methyl orange	10	meso-TiO_2_/	PVDF	25 W UV lamps	720	Higher than 90	[201]
Methylene blue	30	PDA/RGO/Ag_3_PO_4_/	PVDF	200 W incandescent lamp		99.1	[202]
Methyl orange	7.8	TiO_2_ nanoparticles/Dip-Coating	Al_2_O_3_/0.125	300 W high-pressure mercury UV tube		61.2	[203]
RhodamineB	8	MWCNTs/Ag_3_PO_4_/combining electrospinning with in situ Ag_3_PO_4_ forming reaction	PAN	300 W Xe arc lamp	120	96.9	[204]
Methylene blue 4-CP	15	reduced graphene oxide (RGO)/poly(dopamine) (PDA)/Bi1_2_O1_7_Cl_2_/	CA/0.22	500 W long-arc Xe lamp,	160	methylene blue 98, 4-CP 96	[205]
Methylene blue	10	TiO_2_ nanoparticles/Electrospraying TiO_2_ particles	Polyamide-6 nanofiber	300 W Osram Ultra-Vitalux lamp	360	99	[206]
Methylene blue	-	TiO_2_/Magnetron sputtering	PES/0.45		160	70	[207]
Congo red	-	Fe-doped ZnO/rGO/Sol-gel	NF	solar radiation		87	[208]
Methylene blue	3.2	Graphene oxide	PVDF/0.1–0.7	150 W xenon lamp		83.3	[209]
Rhodamine 6G, Rhodamine B	5 to 50	Graphene oxide/direct heating of melamine	PVDF	LED lamp		Rhodamine B 96, Rhodamine 6G 94	[181]
Methylene blue	1	PdTFPP	PVDF/0.2	4.6 W Green and white light emitting diode		83	[210]
Rhodamine B	10	g-C_3_N_4_/RGO/photoreduction	CA/~0.430	Xe lamp	90	90	[211]
Methylene blue	50	nitrogen-doped graphene/TiO_2_/nonsolvent-induced phase-separation	PSF	125W UV lamp, 100 W fluorescent bulb	120	94.6	[212]
Remazol black B	50	g-PAA/ZnO	PVDF/0.45	15 W UV lamp	300	86	[213]
Rhodamine B	5	Bi_2_O_3_/ZnS/	CA/0.11–0.14	200 W xenon light	120	~85	[214]
Acid orange 7	50	SrTiO_3_/TiO_2_/ hydrothermal	CA/0.2	UV lamp		100	[215]
Methyl blue, phenol	methyl blue 5 phenol 3.3	Phosphorus-doped g-C_3_N_4_/ thermal condensation	Al2O3/0.18–0.2	300 W Xe lamp		Phenol 92, methyl blue 90	[82]

**Table 9:** Review on Photocatalytic Membrane degradation of hydrocarbons.

Pollutant	Pollutant Concentration (mg/L)	Photocatalyst/Synthesis Method	Membrane/Pore Size (µm)	Light Source	Time (min)	Degradation (%)	Ref.
Acenaphthene	1–3	TiO_2_	PES	16 W Hg UV-C lamp		Batch process 95.1, Continuous process 80	[216]
Crude oil	100	TiO_2_, BiVO_4_, WO_3_/hydrothermal	PVDF/0.1	14.4W LED strip		89	[217]
Roxarsone		BiOC$l_{0.875}$B$r_{0.125}$	PVDF			100	[218]
propranolol	2	TiO_2_/rGO-TiF	0.160–0.175	UV lamp	60	35	[219]
Bisphenol	10	Ag-doped TiO_2_/ Liquid impregnation—phase inversion	PESf	100 W Xe lamp	270	88	[220]
Naphthalene	5–25	TiO_2_	PES	16 W Hg UV-C lamp	180	batch process 92.8, continuous process 93.1	[183]

**Table 10:** Review on Photocatalytic Membrane degradation of other Pollutants.

Pollutant	Pollutant Concentration (mg/L)	Photocatalyst/Synthesis Method	Membrane/Pore Size (µm)	Setup Configuration	Light Source	Time (min)	Degradation (%)	Ref.
BSA	-	SrTiO_3_–Cr	PVDF	0.2–1 μm	UVA light		Higher than 98	[221]
Oil in water emulsions	-	GO/MCU-C_3_N_4_	PVDF	0.22		30	Higher than 96	[222]
Humic acid	20		Alumina-supported titania/0.657–0.425		mercury lamp with light emission of 1255 μW/cm^2^	180 nin	92	[223]
Oily wastewater	500 and 1000	TiO_2_	Al_2_O_3_/Ceramic membrane	-	UV lamp		90	[224]
Nitrate	10	LiNbO_3_/phase inversion	PES	-	UV light	180	81.82	[225]
Oily wastewater	250–10,000	TiO_2_-P25	PVDF	-	8W UV-A lamp	240	80 TOC degradation	[226]
BSA	1000	GO/TiO_2_/PVP/ solution casting and phase inversion	PVDF	-	UVA irradiation	120	92.5	[227]
Bentazone	10		N–Ti$O_{2}$/PMAA/PVDF/PAN/Loeb-Souriraja	-	UV-light and solar irradiation	180	90.1	[228]
Hexavalent chromium (0Cr (VI))	10	Chitosan-sodium alginate/Fe-doped WO_3_	PES	-	300 W Xe lamp	240	99.9	[186]

## 6. Membrane Fouling in PMRs

Different components of feed, such as organic substances, photocatalysts, colloids, salts, and cells, can have varying effects on system performance [229]. The fouling of PMR membranes is caused by the deposition of feed components on the membrane. Photocatalytic oxidation partially eliminates foulants [230]. Foulant adhesion, pore blocking, the cake layer formation, and temporal and spatial changes of foulant structure during long-term filtration are among factors that promote fouling [230,231]. Photocatalyst nano particles may form microaggregate and deposit on the membrane during filtration. Organic pollutants accumulation in the vicinity of membrane may result in formation of a very thin layer leading to substantial pore clogging and flow rate reduction. Adsorption of organic contaminants on TiO_2_ particles and its composites such as Degussa P25 TiO_2_, Ca alginate polymer/TiO_2_ fibers, nano-structured TiO_2_/silica gel photocatalyst, and titanium tetraisopropoxide in the absence of effective UV absorption may result in formation of a dense cake layer on the membrane surface and further flow rate reduction [232].

Designing non-fouling membranes is highly sought. Zheng et al. [233] combined the cellulose nanocrystals (CNCs) onto Cu-MOF-74 by physically stirring, and then coated the composites on the membrane to enhance the antifouling efficiency of PVDF membranes. CNC/Cu-MOF-74 composite coating on PVDF membrane increased its hydrophilicity, which in turn considerably improved the membrane’s permeability and productivity. They also reported enhanced electrostatic repulsion based on the contact angle test and Zeta-potential measurement. Due to Cu-predominate MOF-74’s antibacterial activity, the CNC/Cu-MOF-74 modified membrane also demonstrated increased antibacterial performance. effective antibacterial performance of composite membrane was attributed to Cu^2+^ release and •OH production.

### 6.1. Reactor Design

Modulating photocatalytic reactions, exchanging catalysts, and degrading pollutants are all easier with slurry PMRs. Although catalyst separation is not required for IPMRs, the catalyst loading cannot be tailored to the feed’s specific composition; higher catalyst loading, larger membrane surface area, and higher-pressure drop are required which increases the reactor volume, energy consumption of pumps, and process cost. Exchanging the catalyst is also a challenging process especially in IPMRs [234,235].

### 6.2. Photocatalyst Loading

Membrane foulants can be reduced and photocatalytic degradation may be accelerated by increasing catalyst loading due to increase reaction surface area. [234]; however, photocatalyst loading has an optimum value for any specific process after which increased opacity of the reaction mixture hinders light absorption by photocatalyst [13,236]. Elevated photocatalyst loading results in reduced foulant degradation which enhances fouling rate on the membrane [237,238].

## 7. Conclusions and Future Perspectives

This review described various hybrid photocatalysis and membrane process designs for removing organic contaminants from water. The main advantage of PMRs is retention and reuse of photocatalyst. The advantages and disadvantages IPMRs and SPMRs, as well as important design/operation parameters were discussed. The performances of a photocatalytic reaction can be improved by utilizing a suspended photocatalyst as opposed to an immobilized one, owing to larger active surface and subsequently improved photocatalyst–substrate interaction. SPMR empowered by air bubbles and effluent flushing appears to be more suitable for treating water and wastewater. Visible light may be utilized as an irradiation source and efficient solar-driven photocatalytic conversion has emerged in recent years. The appropriate selection of the membrane is crucial, as it must have great permeability to the desired product and retain contaminants and photocatalyst to facilitate the rapid removal of the product from the reaction environment. When it comes to treating wastewater, TiO_2_-based PMRs excel because of their excellent separation efficiency and low maintenance requirements. When designing visible-light-operated photocatalysts, it is important to examine the option of employing the sun as a clean, low-cost light source to make the process more environmentally friendly. Advantages of using PMRs for the partial oxidation and reduction of organic matter include (a) extending the lifetime of polymeric membranes with the help of visible light as a source of radiation, and (b) enhancing photocatalyst recovery through the use of novel materials in the synthesis of photocatalyst composites and semiconductor coatings on optical fibers. PMRs utilization has become a mode viable wastewater treatment option upon developments in photovoltaic technology (solar energy conversion) and the use of LED lamps (UV and/or visible).

## Figures and Tables

**Table 1:** Membranes for water decontamination.

Membrane	Material	Manufacturer	Thickness (mm)	Pore Size (μm)	P (bar)	T (K)	Pollutant	Membrane Separation (%)	Ref.
Commercial spiral wound polyamide nano filter	TFC	-	-	-	8	-	COD	98	[52]
AGS reactor, UP and NF	PES, PESH, and PA	-	-		0.5	293.15	COD	51.33, 90.48 and 99.26	[53]
Hollow fiber UF membrane	PVC	-	0.185	0.01–0.1	1	298.15	methyl green dye (MG)	94.79	[54]
NF membrane	piperazine based polyamide, proprietary (cross-linked modified polyacrylonitrile), polyethersulfone	Toray chemical, Koch membrane systems, Nadir	-	0.1	30	-	copper-refining sulfuric acid wastewater	95	[55]
A sheet nano-porous membrane	PAN	Sepro Membranes of USA.	Top layer 0.1–0.5 and Sublayer 100–150	0.01	4	318.15	TSS, TDS, content of oil and grease, COD and BOD5	100, 44.4, 99.9, 80.3 and 76.9	[56]
Multichannel tubular	Ceramic membrane	Jiangsu Jiuwu HiTech Co. Ltd., Nanjing, China		0.05	2	233.15	suspended solid, turbidity, and total phosphorus	100, 99.20, and 80.21,	[57]
SW30 membrane BW30 NF270	PA	-	-	-	69 41 41	318.15	COD and TPh	(95.18, 91.15, 80.11) and (98.02, 96.06, 27.08)	[58]
Difluoride hollow fiber membrane module	PVDF	M/s. TECHNIC, India	-	0.1	0.5–1	-	TOC	64	[59]

**Table 3:** Review on Photocatalytic degradation of pharmaceutical compounds.

Pollutant	Pollutant Concentration (mg·L^−1^)	Photocatalyst	Light Source	Time (min)	Degradation (%)	Ref.
Carbamazepine (CBZ)	2.5	Z-scheme FeS_2_/Fe_2_O_3_ /hexavalent chromium	simulated visible light	30	65	[100]
Penicillin G (PG)	5	TiO_2_/P25	UV-A sunlight (365 nm)	150	72.72	[101]
Pentachlorophenol (PCP)	10	FeNi_3_/SiO_2_/ZnO	Irradiation of solar light	180	92.47	[102]
Ciprofloxacin (CIP)	10	Nano-ZnO	UV	30	96	[103]
Diclofenac (DCL)	1	ZnO	LP Hg (150 W)	30	68	[104]
Sulfamethoxazole	25	TiO_2_	UV	150	61.28	[105]
Sulfamethoxazole	25	WO_3_	UV	150	43.3	[105]
Dexamethasone (DXM)	5	ZrO_2_/WO_3_	halogen	80	100	[106]
Metronidazole (MNZ)	80	illuminated TiO_2_	UV (125 W)	180	96.55	[107]
Levofloxacin	50	BiOCl(25)/BiOBr-Bi_24_O_31_Br_10_(75) type-II nano heterojunction	Halogen (400 W)	180	80.2	[108]
Ofloxacin	50	BiOCl(25)/BiOBr-Bi_24_O_31_Br_10_(75) type-II nano heterojunction	Halogen (400 W)	180	78.3	[108]
Amoxicillin metformin	10	TiO_2_	125 W low-pressure mercury vapor lamp	150	90 98	[109]
Paracetamol	100	La-doped ZnO	fluorescent lamps (20 W)	180	99	[110]
Cefixime	20.5	α-Fe_2_O_3_@TiO_2_	Visible	103	98	[13]
Cefixime	47	g C_3_N_4_/TiO_2_	Visible	113	84	[111]

**Table 4:** Review on Photocatalytic degradation of dye compounds.

Pollutant	Pollutant Concentration (mg/L)	Photocatalyst	Synthesis Method	Light Source	Time (min)	Degradation (%)	Ref.
Acid fuchsine (AF)	120	TiO_2_/ACF	Sol–gel-adsorption	Hg lamp (500 W)	30	77	[116]
4-chlorophenol (4-CP)	15	ZTPG	High-temperature refluxing	UV	180	94.8	[117]
Methylene blue (MB)	20	ZTPG	High-temperature refluxing	UV	90	98.05	[117]
Basic Red 51 (BR51)	1	ZnO	-	sunlight irradiation	330	89.01	[118]
Methylene blue (MB)	9.56	ZnO	-	UV	30	93	[119]
Methyl Orange (MO)	5	TiO_2_/MgO/Chitosan	Hydrogel	UV (125 W)	90	82.4	[120]
Alizarin Red S (ARS)	5	TiO_2_/MgO/Chitosan	Hydrogel	UV (125 W)	90	41.8	[120]
Methyl Orange (MO)	15	Ni@FP + NaBH_4_ (10 mg)	-	UV	5	93.40	[121]
Tartrazine	50	CuCr_2_O_4_	self-combustion	Hg lamp (125 W)	120	99.6	[122]
Acid Brown 98	10	CS-ZnS-NPs	co-precipitation	UV (254 nm)	165	92.6	[123]
Acid Black 234	10	CS-ZnS-NPs	co-precipitation	UV (254 nm)	100	96.7	[123]
Methylene blue (MB)	3.2	MnTiO_3_/TiO_2_	sol-gel	sunlight	240	75	[124]
Tartrazine	10	TiO_2_–Chitosan	-	Sunlight	180	99.37	[125]
1,2-Dichloroethane	250	PAni-TiO_2_	deposition oxidative polymerization	visible	240	88.84	[126]

**Table 5:** Review on Photocatalytic degradation of hydrocarbons.

Pollutant	Pollutant Concentration (mg/L)	Photocatalyst	Synthesis Method	Light Source	Time (min)	Degradation (%)	Ref.
Benzene Toluene Phenol Naphthalene	65	TiO_2_	-	UV (400 W)	90	92 98.8 91.5 93	[130]
Paraffin	500	TiO_2_/SiO_2_ thin film	sol-gel	UV	180	85	[131]
BTEX TPHs	60.8	TiO_2_	-	solar light	240	>70	[132]
Naphthalene	50	ZnO/Ag/GO nanocomposit	-	Xe lamp (250 W)	20	80	[133]
BTEX	600	γ-Fe_2_O_3_ nanoparticle	-	UV light (100 W)	90	97	[134]
Naphthalene	40	Calcinated Fe-doped ZnO/PVA nanofibers	-	UV light (16 W)	360	96	[135]
Chrysene	2	Fe_2_O_3_@ZnHCF nanocubes	-	sunlight	1440	92	[136]
Benzene, toluene and xylenes (BTX) and gasoline-contaminated waters	20	TiO_2_-Fenton system	-	medium-pressure mercury vapor lamp (125 W)	90	75	[137]
Formaldehyde	700	MIL-100(Fe)	solvothermal	Visible	119	93	[138]

**Table 6:** Review on Photocatalytic degradation of other pollutants.

Pollutant	Pollutant Concentration (mg/L)	Photocatalyst	Synthesis Method	Light Source	Time (min)	Degradation (%)	Ref.
Bisphenol-A (BPA)	50	ZnFe_2_O_4_/leaf extract of Azarachita indica	-	sunlight	720	92	[142]
Bisphenol-A (BPA)	50	CoFe_2_O_4_/leaf extract of Azarachita indica	-	sunlight	720	89	[142]
Bisphenol-A (BPA)	50	Fe_2_O_3_/leaf extract of Azarachita indica	-	sunlight	720	70	[142]
Bisphenol-A (BPA)	50	ZnO/leaf extract of Azarachita indica	-	sunlight	720	68	[142]
Bisphenol-A (BPA)	50	Co_3_O_4_/leaf extract of Azarachita indica	-	sunlight	720	54	[142]
Para-aminobenzoic acid	20	TiO_2_ P25	-	UV (9 W)	120	>80	[143]
toluidine blue o, safranin o, falcon carboxylic acid, Hexavalent chromium Cr	5	ZnO	-	UV light (250 W)	190, 310, 260, 300	94, 87, 92, 68	[144]
Hexavalent chromium Cr (VI) bisphenol A (BPA)	10	magnetic 3D-TiO_2_@HPGA	solvothermal process	low pressure mercury vapor lamps (8 W)	140 240	100 90	[145]
Clofibric acid (CA)	2	g-C_3_N_4_	-	Xe-lamp (350 W)	<50	46.8	[146]
Clofibric acid (CA)	2	P25	-	Xe-lamp (350 W)	<50	56.8	[146]
Clofibric acid (CA)	2	g-C_3_N_4_/P25 (8 wt%)	-	Xe-lamp (350 W)	<50	85.4	[146]
Atrazine	100	Cu-ZnO/g-C_3_N_4_ Z-direct scheme	-	UV	120	90	[147]
Herbicide glyphosate	100	BiOBr/Fe_3_O_4_ nanocomposites	Chemical co-precipitation method	Xe lamp (500 W)	60	97	[148]
Diuron herbicide	25	g-C_3_N_4_/N-doped CeO_2_ composite	-	Xe lamp (1500 W)	120	46	[149]
Gramoxone herbicide	10	TiO_2_ hollow fibers	-	UV lamp (6 W)	480	<50	[150]
Chlorpyrifos	2	CeO_2_/TiO_2_/SiO_2_	sonophotocatalytic	Visible	150	90.8	[151]

**Table 7:** Review on Photocatalytic Membrane degradation of pharmaceutical compounds.

Pollutant	Pollutant Concentration (mg/L)	Photocatalyst/Synthesis Method	Membrane/Pore Size (µm)	Light Source	Time (min)	Degradation (%)	Ref.
Chloramphenicol	50	TiO_2_ doped hydroxyapatite/hydrothermal	polysulfone (PSF)/0.003	UV Light	120	61.59	[160]
Pharmaceutical industry wastewater	TDS 4740 COD 17360	Cerium fluoride (CeF)/Phase inversion by immersion precipitation technique	polysulfone (PSF)	24 W UV lamp		97	[161]
Triclosan	10	bismuth vanadate/polyethyleneimine blended in polyethersulphone		300 W Xenon Lamp		86	[162]
Sulfamethoxazole	10	mesoporous graphitic carbon nitride/TiO_2_		300 W ozone free xenon lamp		49	[163]
Sulfadiazine	12	Goethite (α-FeOOH)/precipitation	0.14	UVL214W lamp		70 (no H_2_O_2_) and 99 (with H_2_O_2_)	[164]
Diclofenac	10	N, S-CQDs/TiO_2_	polysulfone (PSF)	light (40 W), visible light (12 W)	150	62.3	[165]
Oxytetracycline	20	TiO_2_	UF membrane	UVC lamp (16 W)		52 DOC removal	[166]
Diphenhydramine	-	graphene oxide-TiO_2_	Mixed cellulose ester (MCE)	UV/Vis and visible light irradiation		73	[167]
Chlorhexidine Digluconate	-	TiO_2_	PES and PVC-PAN	1500 W solar simulator		40	[168]
Diclofenac	2	Ti$O_{2}$	PVDF/0.04	4 × 24 W black Light		99.5	[169]
Diclofenac and ibuprofen	Diclofenac sodium salt 25, ibuprofen sodium salt 100	TiO_2_	PES and PVDF/0.22	7.6 mW/cm^2^ UVA lamp	120	For PES 68, For PVDF 55	[170]
Diclofenac (DCF), ibuprofen (IBU) and naproxen (NAP)	100 μg d$m^{-3}$	TiO_2_	Polypropylene (PP)/0.2	16 W UVC germicidal lamp		DCF100, IBU 73, NAP 90	[171]
furosemide, ranitidine, ofloxacine, phenazone, naproxen, carbamazepine	10	TiO_2_	different NF membranes (PES, PSF, PAN)	125 W medium pressure Hg lamp	120	furosemide 80, ranitidine 50, Naproxen 90	[172]
tetracycline	5	Au/Ag/Ti$O_{2}$	CA/0.213	Xe lamp		90	[173]
Diclofenac	0.05	TiO_2_	PVDF/0.03	52 W UV-C power		100	[174]
Tetracycline	10	Au-TiO_2_	PDA-PVDF/0.22	Xenon lamp	120	90	[175]
Diclofenac	50 for batch 20 for continuous	N-doped TiO_2_	MF membrane 0.1, RO membrane 0.0001–0.001	5 × visible 250 W lamps	150–180	97.66	[84]
Ciprofloxacin	10	sulfonated graphene oxide/ZnO	PES/0.011	150 W UV	240	95.1	[176]
Pharmaceutical industry wastewater	TDS 4740 COD 17360	ZrO_2_– SnO_2_/sol-gel	PS	24 W UV lamp		90	[177]
Tetracycline	10	ZnI$n_{2}S_{4}$	PVDF/0.3	150 W halogen tungsten lamp		50	[178]
Carbamazepine	1	α-A$l_{2}O_{3}$ coated with N-doped Ti$O_{2}$/Sol-gel	0.2–0.8	300 W ozone-free xenon arc lamp		90	[179]

## Data Availability

All data generated or analyzed during this study are included in this published article.

## Abbreviations

AF	Acid fuchsine	MRs	Membrane reactors
ANA	acenaphthene	MWCO	molecular weight cut-offs
AOPs	advanced oxidation processes	MS	multiple sclerosis
ARS	Alizarin red S	NAP	naphthalene
BPA	Bisphenol-A	NF	nanofiltration
BR51	Basic Red 51	PANI	Polyaniline
BSA	bovine serum albumin	PCP	Pentachlorophenol
BTEX	benzene, toluene, ethylbenzene, and xylene	PdTFPP	(pentafluorophenyl)-21H, 23H-porphine palladium (II)
CA	Clofibric acid	PE	polyethylene
CB	Conduction bond	PECM	photoelectrocatalytic membrane filtration
CBZ	carbamazepine	Penicillin G	Benzylpenicillin
CeF	Cerium fluoride	PES	Polyethersulfone
CFX	Cefixime	PHE	phenanthrene
CIP	Ciprofloxacin	pHpzc	point of zero charge
CNCs	cellulose nanocrystals	PMRs	photocatalytic membrane reactors
COS	carbon oxidation state	PP	Polypropylene
DCF	diclofenac	PS	Phosphatidylserine
DCL	Diclofenac	PVDF	polyvinylidene fluoride
DXM	Dexamethasone	RB5	Reactive Black 5
$E_{a}$	band-gap energy	RO	Reverse osmosis
$e_{\mathrm{CB}}^{-}$	conduction band electron	ROS	reactive oxygen species
HPC	heterogeneous photocatalysis	SPMR	Suspended photocatalytic membrane reactors
hv	irradiated photons	TMP	transmembrane pressure
$h_{\mathrm{VB}}^{+}$	valence band hole	TPHs	total petroleum hydrocarbons
IBU	ibuprofen	UF	ultrafiltration
IPMR	immobilized photocatalytic membrane reactors	UV	visible light
MB	methylene blue	VB	Valance band
MF	microfiltration	ZnONSt	zinc oxide nano stars
MIL-53(Fe)	Matériaux de l′Institut Lavoisier	ZTPG	ZnO tetrapod-reduced graphene oxide
MNZ	Metronidazole		
MO	Methyl Orange		
MOF	Metal–organic framework		
AF	Acid fuchsine		
ANA	acenaphthene		
AOPs	advanced oxidation processes		
ARS	Alizarin red S		
BPA	Bisphenol-A		
BR51	Basic Red 51		
BSA	bovine serum albumin		
BTEX	benzene, toluene, ethylbenzene, and xylene		
CA	Clofibric acid		
CB	Conduction bond		
CBZ	carbamazepine		
CeF	Cerium fluoride		
CFX	Cefixime		
